# Assessing the Quality of Patient Education Materials on Cardiac Catheterization From Artificial Intelligence Chatbots: An Observational Cross-Sectional Study

**DOI:** 10.7759/cureus.69996

**Published:** 2024-09-23

**Authors:** Benjamin J Behers, Christoph A Stephenson-Moe, Rebecca M Gibons, Ian A Vargas, Caroline N Wojtas, Manuel A Rosario, Djhemson Anneaud, Profilia Nord, Karen M Hamad, Joel F Baker

**Affiliations:** 1 Department of Internal Medicine, Sarasota Memorial Hospital, Sarasota, USA; 2 Department of Clinical Sciences, Florida State University College of Medicine, Tallahassee, USA

**Keywords:** cardiac catheterization, chatgpt, google gemini, meta ai, microsoft copilot, patient education

## Abstract

Background

Health literacy empowers patients to participate in their own healthcare. Personal health literacy is one’s ability to find, understand, and use information/resources to make well-informed health decisions. Artificial intelligence (AI) has become a source for the acquisition of health-related information through large language model (LLM)-driven chatbots. Assessment of the readability and quality of health information produced by these chatbots has been the subject of numerous studies to date. This study seeks to assess the quality of patient education materials on cardiac catheterization produced by AI chatbots.

Methodology

We asked a set of 10 questions about cardiac catheterization to four chatbots: ChatGPT (OpenAI, San Francisco, CA), Microsoft Copilot (Microsoft Corporation, Redmond, WA), Google Gemini (Google DeepMind, London, UK), and Meta AI (Meta, New York, NY). The questions and subsequent answers were utilized to make patient education materials on cardiac catheterization. The quality of these materials was assessed using two validated instruments for patient education materials: DISCERN and the Patient Education Materials Assessment Tool (PEMAT).

Results

The overall DISCERN scores were 4.5 for ChatGPT, 4.4 for Microsoft Copilot and Google Gemini, and 3.8 for Meta AI. ChatGPT, Microsoft Copilot, and Google Gemini tied for the highest reliability score at 4.6, while Meta AI had the lowest with 4.2. ChatGPT had the highest quality score at 4.4, while Meta AI had the lowest with 3.4. ChatGPT and Google Gemini had Understandability scores of 100%, while Meta AI had the lowest with 82%. ChatGPT, Microsoft Copilot, and Google Gemini all had Actionability scores of 75%, while Meta AI had one of 50%.

Conclusions

ChatGPT produced the most reliable and highest quality materials, followed closely by Google Gemini. Meta AI produced the lowest quality materials. Given the easy accessibility that chatbots provide patients and the high-quality responses that we obtained, they could be a reliable source for patients to obtain information about cardiac catheterization.

## Introduction

Health literacy is integral in empowering patients to participate in their own healthcare. Personal health literacy is one’s ability to find, understand, and use information/resources to make well-informed health decisions, and is a central part of the Healthy People 2030 initiative [1]. A major element of personal health literacy is the ability to understand and utilize health-related information from different formats [2]. In recent years, increased access to and expansion of the internet has resulted in website-based patient education materials replacing paper handouts obtained directly from healthcare providers. Assessments of the quality and readability of these online patient education materials have been the subject of hundreds of studies to date.

Within the rapidly expanding digital realm, artificial intelligence (AI) has become a potential resource for the acquisition of health-related information. This increased use of AI for health education has corresponded to the development of large language model (LLM)-driven chatbots [3]. Users can ask chatbots questions and receive information compiled from online sources [3]. More recently, AI has become implemented into search engines, making it more accessible for asking health-related questions. As with websites, numerous studies have been performed to assess the quality and readability of these AI chatbot-generated patient education materials. However, given the novelty of this technology, many topics have yet to be investigated.

In a previous study, we assessed the readability of patient education materials on cardiac catheterization generated through four AI chatbots: ChatGPT (OpenAI, San Francisco, CA), Microsoft Copilot (Microsoft Corporation, Redmond, WA), Google Gemini (Google DeepMind, London, UK), and Meta AI (Meta, New York, NY)[4]. We found that these materials had reading grade levels at the high school or college reading level, far exceeding the sixth-grade level recommended by the American Medical Association and National Institutes of Health [4]. While readability is an important aspect of health education materials, the quality of the information provided is equally as important. This current study seeks to build upon our previous findings and assess the quality of health education materials on cardiac catheterization from AI chatbots.

## Materials and methods

We asked a set of 10 questions to four different chatbots: ChatGPT Version 3.5, Microsoft Copilot, Google Gemini 1.0 Pro, and Meta AI Version 24.0. These 10 questions can be seen in Table 1. The questions and answers from each chatbot were copied into a Microsoft Word (Version 15.30) document to create patient education material akin to what can be found on health information websites.

**Table 1:** The 10 questions asked to each chatbot

Questions
What is cardiac catheterization?
Why is cardiac catheterization needed?
What happens during cardiac catheterization?
What are the benefits of cardiac catheterization?
What are the risks of cardiac catheterization?
How do I prepare for cardiac catheterization?
What happens before cardiac catheterization?
What happens after cardiac catheterization?
What type of results do you get from cardiac catheterization?
When should you call your doctor after cardiac catheterization?

The quality of these patient education materials was then assessed using two validated instruments: DISCERN and the Patient Education Materials Assessment Tool (PEMAT) [5,6]. The first two authors (BJB and CASM) independently screened these materials, assigning ratings as outlined by the two instruments. Disagreements were resolved via discussion, with the last author (JFB) available if consensus was unable to be achieved.

The DISCERN instrument assesses the quality of written health information using 16 questions across three sections: reliability of the publication (Questions 1-8), quality of information on treatment choices (Questions 9-15), and overall rating (Question 16) [5]. Each question is scored one to five, with a score of one corresponding to “No” and five corresponding to “Yes,” while three represents “Partially” [5]. Given that sources are not generally provided by AI chatbots, we excluded DISCERN questions that asked about these. This excluded three questions from the reliability of the publication section. We also calculated the overall rating by taking an average of the ratings for the rest of the questions, as opposed to simply rating this from one to five. The modified DISCERN that we used for this study can be seen in Table 2.

**Table 2:** The modified DISCERN instrument used in this study

Section	Question
Section 1: Is the publication reliable?	1. Are the aims clear?
	2. Does it achieve its aims?
	3. Is it relevant?
	4. Is it balanced and unbiased?
	5. Does it refer to areas of uncertainty?
Section 2: How good is the quality of information on treatment choices?	6. Does it describe how each treatment works?
	7. Does it describe the benefits of each treatment?
	8. Does it describe the risks of each treatment?
	9. Does it describe what would happen if no treatment is used?
	10. Does it describe how the treatment choices affect overall quality of life?
	11. Is it clear that there may be more than one possible treatment choice?
	12. Does it provide support for shared decision-making?

PEMAT is a systematic method to determine whether health education information is understandable and actionable for patients [6]. It consists of 24 questions for printable materials that are scored zero for “Disagree” and one for “Agree,” with certain questions having the option of N/A for “Not Applicable” [6]. Scores are assigned separately for the Understandability and Actionability domains by taking the total number of points divided by the total possible points (excluding questions scored N/A) and multiplying by 100% [6]. The PEMAT questions can be seen in Table 3.

**Table 3:** The Patient Education Materials Assessment Tool (PEMAT) instrument questions

Domain	Topic	Item
Understandability	Content	1. The material makes its purpose completely evident.
		2. The material does not include information or content that distracts from its purpose.
	Word choice and style	3. The material uses common, everyday language.
		4. Medical terms are used only to familiarize audience with the terms. When used, medical terms are defined.
		5. The material uses the active voice.
	Use of numbers	6. Numbers appearing in the material are clear and easy to understand.
		7. The material does not expect the user to perform calculations.
	Organization	8. The material breaks or “chunks” information into short sections.
		9. The material’s sections have informative headers.
		10. The material presents information in a logical sequence.
		11. The material provides a summary.
	Layout and design	12. The material uses visual cues (e.g., arrows, boxes, bullets, bold, larger font, highlighting) to draw attention to key points.
	Use of visual aids	13. The material uses visual aids whenever they could make content more easily understood (e.g., illustration of healthy portion size).
		14. The material’s visual aids reinforce rather than distract from the content.
		15. The material’s visual aids have clear titles or captions.
		16. The material uses illustrations and photographs that are clear and uncluttered.
		17. The material uses simple tables with short and clear row and column headings.
Actionability		18. The material clearly identifies at least one action the user can take.
		19. The material addresses the user directly when describing actions.
		20. The material breaks down any action into manageable, explicit steps.
		21. The material provides a tangible tool (e.g., menu planners, checklists) whenever it could help the user take action.
		22. The material provides simple instructions or examples of how to perform calculations.
		23. The material explains how to use charts, graphs, tables, or diagrams to take actions.
		24. The material uses visual aids whenever they could make it easier to act on the instructions.

## Results

ChatGPT had the highest DISCERN scores, overall and for both sections, of 4.5, 4.6, and 4.4, respectively. Microsoft Copilot and Google Gemini were tied for second with an overall score of 4.4 and section scores of 4.6 and 4.3, respectively. Meta AI had the lowest DISCERN scores across all three, with an overall score of 4.2 and scores of 3.4 and 3.8 on Sections 1 and 2, respectively. Higher scores across Section 1 indicate better reliability of the publication, while higher scores in Section 2 indicate better quality of information provided on treatment choices. Our results indicate that ChatGPT, Microsoft Copilot, and Google Gemini all provide equally reliable information, but ChatGPT provides slightly better quality information. The DISCERN scores for each chatbot can be found in Table 4.

**Table 4:** Average DISCERN scores for each chatbot for Section 1 (reliability of the publication), Section 2 (quality of information), and overall

Chatbot	Section 1 average (reliability)	Section 2 average (quality)	Overall
ChatGPT	4.6	4.4	4.5
Microsoft Copilot	4.6	4.3	4.4
Google Gemini	4.6	4.3	4.4
Meta AI	4.2	3.4	3.8

We further investigated specific differences between chatbots in scores to the DISCERN questions. In Section 1, none of the chatbots received a perfect score of five on Question #5, “Does it refer to areas of uncertainty?” Using the DISCERN instrument, ChatGPT, Microsoft Copilot, and Google Gemini received scores of three or “Partially” due to their failure to mention gaps in knowledge or differences in expert opinion. However, they did a sufficient job of discussing that individual patients will have different risk factors, procedures, and outcomes, which is the other aspect of this question. Meta AI performed lower on this same question with a score of two due to its failure to mention knowledge gaps, as well as providing less emphasis on the variations to be expected between patients. The only other question in Section 1 that did not receive a score of five was Question #3, “Is it relevant?” Meta AI received a score of four on this question due to its failure to recognize and address questions that readers might ask.

**Figure 1:**
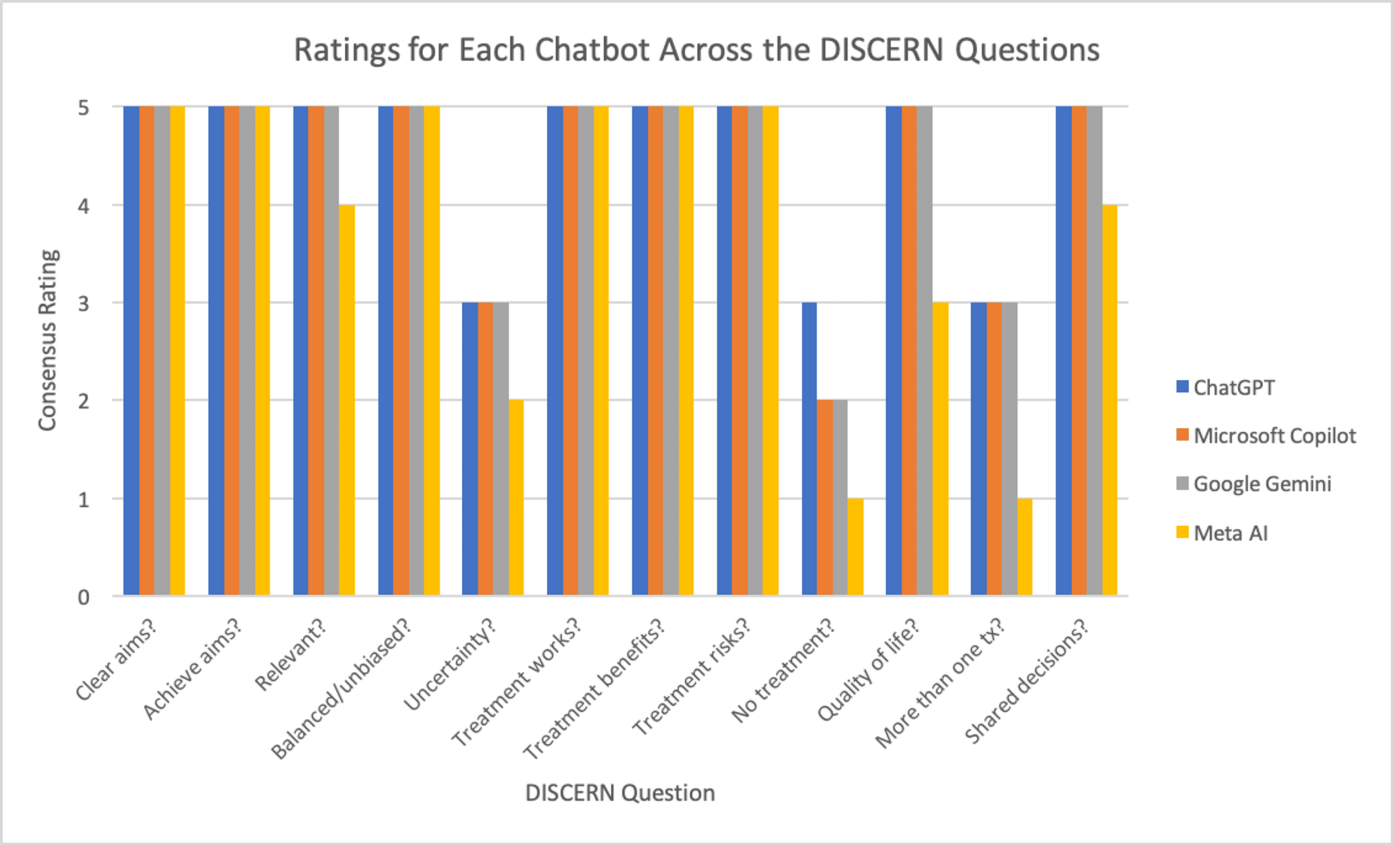
Individual scores for each chatbot across the 12 DISCERN questions Clear aims?: Q1. Are the aims clear?; Achieve aims?: Q2. Does it achieve its aims?; Relevant?: Q3. Is it relevant?; Balanced/unbiased?: Q4. Is it balanced and unbiased?; Uncertainty?: Q5. Does it refer to areas of uncertainty?; Treatment works?: Q6. Does it describe how each treatment works?; Treatment benefits?: Q7. Does it describe the benefits of each treatment?; Treatment risks?: Q8. Does it describe the risks of each treatment?; No treatment?: Q9. Does it describe what would happen if no treatment is used?; Quality of life?: Q10. Does it describe how the treatment choices affect the overall quality of life?; More than one treatment?: Q11. Is it clear that there may be more than one possible treatment choice?; Shared decisions?: Q12. Does it provide support for shared decision-making?

In Section 2, all four chatbots received scores of five on Questions #6-8, which judge whether the materials effectively describe each treatment, its benefits, and its risks. The lowest scores were seen with Question #9, “Does it describe what would happen if no treatment is used?” ChatGPT received a high score of three due to its mention that cardiac catheterization can help reduce the risk of heart attacks, heart failure, and other serious complications by accurately diagnosing and treating heart conditions. However, we could only score this a three because of its failure to explicitly describe what would happen if no treatment was used. On the other hand, Meta AI received a score of one due to its failure to mention or even imply the consequences of no treatment. Microsoft Copilot and Google Gemini received scores of two due to their implications of what would happen without treatment, but failure to explicitly state it and lesser emphasis than we saw with ChatGPT. The other question that none of the chatbots received a score of five on was Question #11, “Is it clear that there may be more than one possible treatment choice?” ChatGPT, Microsoft Copilot, and Google Gemini all received scores of three due to their mention of open heart surgery as an alternative. However, they did not elaborate on heart surgery and failed to mention whether that was the only alternative. Meta AI received a score of one on this question for its failure to mention heart surgery as an alternative. Meta AI also received a score of three on Question #10, “Does it describe how the treatment choices affect the overall quality of life?” due to its vague responses about what to expect after cardiac catheterization and failure to explicitly mention the quality of life outcomes. It also received a score of four on Question #12, “Does it provide support for shared decision-making?” due to it being more direct than the others and its failure to explicitly lay out that patients should collaborate with their healthcare providers when considering cardiac catheterization. Differences in the scores for each chatbot across the 12 DISCERN questions can be seen in Figure 1. 

ChatGPT and Google Gemini had the same PEMAT Understandability and Actionability scores of 100% and 75%, respectively. Microsoft Copilot also had an Actionability score of 75% but a slightly lower Understandability score of 92%. The only difference in Understandability for these three chatbots was Item #11 and Microsoft Copilot’s failure to provide a summary. Additionally, all three chatbots received a score of 0 for Item #21 for failure to provide a tangible tool to help the user take action. Meta AI had the lowest scores, with an Understandability score of 82% and an Actionability score of 50%. Meta AI missed points on Understandability because of Items #4 and #11, which failed to define medical terms consistently and lacked a summary, respectively. It missed points on Actionability due to Items #22-23 and its failure to break actions into manageable, explicit steps, as well as its failure to provide a tangible tool for action. The PEMAT scores for each chatbot can be found in Table 5.

**Table 5:** Patient Education Materials Assessment Tool (PEMAT) scores for each chatbot broken down by Understandability and Actionability domains Overall percentage scores are provided with parentheses denoting the number of items agreed to as the numerator and the total number of items answered as the denominator, i.e., items scored “Not Applicable” were not included in the denominator.

Chatbot	Understandability	Actionability
ChatGPT	100% (12/12)	75% (3/4)
Microsoft Copilot	92% (11/12)	75% (3/4)
Google Gemini	100% (11/11)	75% (3/4)
Meta AI	82% (9/11)	50% (2/4)

Items #13-17, which deal with the use of visual aids, were labeled N/A for all four chatbots due to the lack of visual aids found in chatbot responses. This resulted in a maximum number of Understandability questions of 12. Google Gemini and Meta AI both only had 11 questions because Item #6 was scored N/A due to their lack of use of numbers. Similarly, Items #22-24 also received scores of N/A for all four chatbots due to the lack of calculations to perform, as well as further questions on the use of visual aids. This resulted in four Actionability questions, which were applicable to all four chatbots.

## Discussion

Overall, ChatGPT appears to provide the highest quality patient education materials on cardiac catheterization. Our results suggest that ChatGPT’s materials are the most reliable, understandable, and actionable and contain the highest quality materials produced by the four chatbots. Google Gemini provided similarly reliable, understandable, and actionable materials but of slightly lower quality. Conversely, Meta AI was found to provide the lowest quality patient education materials across all four measures. Our study suggests that three of these four chatbots, excluding Meta AI, are capable of providing high-quality patient education materials on cardiac catheterization based on our analysis using DISCERN and PEMAT, two validated tools specifically designed to assess the quality of patient education materials [5,6]. 

Our findings that AI chatbots produce high-quality patient education materials are reassuring, given the increasing rate at which patients are utilizing chatbots for health information. As mentioned, health literacy allows patients to better participate in their own care and has been associated with better health outcomes [1,7]. Improvement in health literacy is especially vital in patients with cardiovascular diseases, as poor health literacy has been associated with increased mortality, increased hospital admissions, and decreased quality of life [8,9]. However, for patient education materials to positively impact health literacy, they need to be both readable and accurate. Prior studies, including our own on cardiac catheterization, have shown that chatbot-generated materials are written significantly above the recommended sixth-grade level, a significant limitation to the furtherance of health literacy [4]. However, other studies have shown the ability of AI chatbots to simplify information when prompted to, albeit at the possible expense of accuracy [4,10].

While there seems to be consensus on the readability of these materials, studies are conflicted on the quality and accuracy. A recent systematic review on the role of ChatGPT in cardiology concluded that while there may be some benefit to its use in patient education, this is limited by inaccuracies in its outputs, incomplete answers, and the inability to provide the most up-to-date information [11]. A study comparing ChatGPT’s heart failure education to that of national cardiology institutes found that ChatGPT was less readable and had the lowest PEMAT Actionability score of any material [12]. Furthermore, a study comparing chatbots to traditional patient information leaflets (PILs) on local anesthesia in eye surgery found the traditional leaflets to be superior in readability, accuracy, and completeness [13]. However, another study by the same authors found no differences in accuracy, completeness, understandability, and actionability between PILs and ChatGPT patient education materials on chronic pain medications [14]. Additionally, another study found that ChatGPT-4 used multiple academic sources to answer queries about the Latarjet procedure compared to Google Search Engine using single-surgeon and large medical practice websites, although the clinical relevance and accuracy of the information were not significantly different [15]. This highlights the strength of LLM chatbots in compiling and referencing high-quality sources when providing their responses.

As we saw in our study, each chatbot is different and provides varying levels of information quality with its responses. Given that ChatGPT is the most common chatbot, many studies focus solely on it, but there are studies similar to ours that compare multiple chatbots. A comparison of responses from five chatbots (ChatGPT-4, Claude, Mistral, Google PaLM, and Grok) to the most frequently asked questions (FAQs) on kidney stones found Grok to be the easiest to read and ChatGPT the hardest, while Claude had the best text quality [16]. Another study assessing the responses of ChatGPT, Bard, Gemini, Copilot, and Perplexity in responses to FAQs about palliative care found Perplexity and Gemini to be the highest quality [17]. However, another study assessing the responses of ChatGPT-3.5 and ChatGPT-4 to FAQs by patients undergoing deep brain stimulation found significantly better and more complete responses by ChatGPT-4, as determined by experts in the field [18]. This highlights the variability in the abilities of the different chatbots to provide quality patient education materials, even among different versions of the same instrument. Given the ever-changing nature of chatbots, this variability is likely also constantly changing and will be difficult to predict.

This study is not without its limitations, the most notable of which is that the DISCERN and PEMAT instruments have not been validated for use on AI chatbot-generated patient education materials. Furthermore, the questions on the DISCERN tool that we excluded dealt with the quality and reliability of sources used to compile the information. While it is known that these chatbots are trained on large amounts of data across the internet and have been shown to provide reliable and factually correct responses, the exact sources of the information provided cannot be determined. This limits our ability to confidently promote the quality of these materials, and our results should thus be taken with caution. Additionally, the use of a modified DISCERN questionnaire, as well as the number of items that were deemed “Not Applicable” on the PEMAT, makes comparison of the quality of these materials to prior studies challenging. We also only asked each question once to the chatbots, so our results are only reflective of that single point in time and cannot account for any updates to these models or differences in responses to the questions that asking multiple times may have elicited. Finally, only two authors assessed the quality of the responses and disagreements in the initial screening, which were solved through discussion, lending the possibility of bias through groupthink and confirmation bias. Future studies should utilize more authors to independently rate the materials and calculate inter-rater reliability to limit this bias and achieve a better understanding of the true quality of these materials.

## Conclusions

To our knowledge, this is the first study to assess the quality of patient education materials on cardiac catheterization developed by AI chatbots. ChatGPT produced the most reliable and highest quality materials, followed closely by Google Gemini. Meta AI produced the lowest quality materials. Given the easy accessibility that chatbots provide patients and the high-quality responses that we obtained, they could be a reliable source for patients to obtain information about cardiac catheterization. However, future studies should compare the information obtained from chatbots to conventional methods, such as PILs and websites, to determine which source provides the highest quality information.

## References

[1] (2024). Health Literacy in Healthy People 2030. https://health.gov/healthypeople/priority-areas/health-literacy-healthy-people-2030.

[2] Liu C, Wang D, Liu C (2020). What is the meaning of health literacy? A systematic review and qualitative synthesis. Fam Med Community Health.

[3] Golan R, Reddy R, Ramasamy R (2024). The rise of artificial intelligence-driven health communication. Transl Androl Urol.

[4] Behers BJ, Vargas IA, Behers BM, Rosario MA, Wojtas CN, Deevers AC, Hamad KM (2024). Assessing the readability of patient education materials on cardiac catheterization from artificial intelligence chatbots: an observational cross-sectional study. Cureus.

[5] (2024). The DISCERN Instrument. http://www.discern.org.uk/index.php.

[6] Shoemaker SJ, Wolf MS, Brach C (2014). Development of the Patient Education Materials Assessment Tool (PEMAT): a new measure of understandability and actionability for print and audiovisual patient information. Patient Educ Couns.

[7] Tepe M, Emekli E (2024). Assessing the responses of large language models (ChatGPT-4, Gemini, and Microsoft Copilot) to frequently asked questions in breast imaging: a study on readability and accuracy. Cureus.

[8] Berkman ND, Sheridan SL, Donahue KE, Halpern DJ, Crotty K (2011). Low health literacy and health outcomes: an updated systematic review. Ann Intern Med.

[9] Kanejima Y, Shimogai T, Kitamura M, Ishihara K, Izawa KP (2022). Impact of health literacy in patients with cardiovascular diseases: a systematic review and meta-analysis. Patient Educ Couns.

[10] Sudharshan R, Shen A, Gupta S, Zhang-Nunes S (2024). Assessing the utility of ChatGPT in simplifying text complexity of patient educational materials. Cureus.

[11] Sharma A, Medapalli T, Alexandrou M, Brilakis E, Prasad A (2024). Exploring the role of ChatGPT in cardiology: a systematic review of the current literature. Cureus.

[12] Anaya F, Prasad R, Bashour M, Yaghmour R, Alameh A, Balakumaran K (2024). Evaluating ChatGPT platform in delivering heart failure educational material: a comparison with the leading national cardiology institutes. Curr Probl Cardiol.

[13] Gondode P, Duggal S, Garg N, Lohakare P, Jakhar J, Bharti S, Dewangan S (2024). Comparative analysis of accuracy, readability, sentiment, and actionability: artificial intelligence chatbots (ChatGPT and Google Gemini) versus traditional patient information leaflets for local anesthesia in eye surgery. Br Ir Orthopt J.

[14] Gondode P, Duggal S, Garg N, Sethupathy S, Asai O, Lohakare P (2024). Comparing patient education tools for chronic pain medications: artificial intelligence chatbot versus traditional patient information leaflets. Indian J Anaesth.

[15] Oeding JF, Lu AZ, Mazzucco M (2024). ChatGPT-4 performs clinical information retrieval tasks using consistently more trustworthy resources than does Google Search for queries concerning the Latarjet procedure. Arthroscopy.

[16] Şahin MF, Topkaç EC, Doğan Ç, Şeramet S, Özcan R, Akgül M, Yazıcı CM (2024). Still using only ChatGPT? The comparison of five different artificial intelligence chatbots' answers to the most common questions about kidney stones. J Endourol.

[17] Hancı V, Ergün B, Gül Ş, Uzun Ö, Erdemir İ, Hancı FB (2024). Assessment of readability, reliability, and quality of ChatGPT®, BARD®, Gemini®, Copilot®, Perplexity® responses on palliative care. Medicine (Baltimore).

[18] Oliveira AL, Coelho M, Guedes LC, Cattoni MB, Carvalho H, Duarte-Batista P (2024). Performance of ChatGPT 3.5 and 4 as a tool for patient support before and after DBS surgery for Parkinson’s disease. Neurol Sci.

